# Utility of Three-Dimensional and Four-Dimensional Transesophageal Echocardiography in Decision-Making in a Patient with Iatrogenic Left Ventricle–to–Right Atrium Shunt (Gerbode Defect)

**DOI:** 10.1016/j.case.2022.12.006

**Published:** 2023-01-27

**Authors:** Umang Gupta, Mohsen Karimi

**Affiliations:** University of Iowa, Stead Family Children's Hospital, Iowa City, Iowa

**Keywords:** 3D echocardiography, Structural heart disease, Device closure, LV-RA shunt, Gerbode defect

## Abstract

• Two-dimensional echocardiography is the workhorse of cardiovascular imaging.
• Three-dimensional echocardiography can improve diagnostic capability.
• In structural and congenital heart disease, 3D echo can be extremely useful.
• Three-dimensional echo should be used more often in cardiac interventional procedures.

## Graphical abstract

**Figure:**
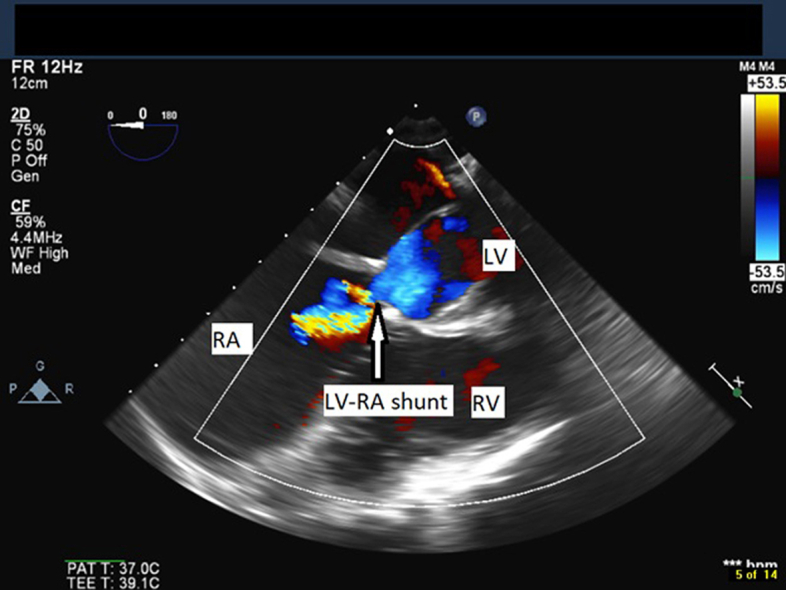

## Introduction

Since its first introduction, the role of three-dimensional (3D) echocardiography in diagnosing structural and congenital cardiac defects has been identified. However, its adaptation for congenital and structural defects has been slow. This is primarily driven by a lack, until recently, of dedicated pediatric 3D transesophageal echocardiography (TEE) probes, the relatively small size of the primary target population with these defects, no learning curriculum, limitations of “on-the-cart” software, and the length of time needed to process more complex lesions. With advancement in probe technology and software quality, there has been a renewed interest in using 3D echocardiography in real time during procedures in patients with structural and congenital heart diseases. It has been found to provide invaluable information during interventional procedures that helps with decision-making.

In this report we describe a case of an iatrogenic left ventricle– (LV) to–right atrium (RA) shunt (Gerbode defect) where the real-time 3D (4D) echocardiogram provided additional information that was not available through two-dimensional (2D) echocardiography and that impacted management decisions.

## Case Presentation

A 43-year-old man with a known diagnosis of Noonan's spectrum disorder/RASopathy, secondary to pathogenic variant in SOS1 (c.2536 G>A; p. Glu846Lys), was initially referred to our tertiary-care center for care of their complex multisystem disease. A cardiac evaluation was ordered as part of the workup and was concerning for a right atrial mass. Cardiovascular magnetic resonance (CMR) imaging was performed to further delineate the mass and for tissue characterization. The CMR showed a pedunculated mass attached to the atrial septum in the RA with characteristics consistent with myxoma. The patient was also noted to have an additional small atrial septal defect (ASD). They subsequently underwent ASD repair and RA mass excision. After an uneventful postoperative course, they were discharged. However, during follow-up they were noted to have developed an LV-to-RA shunt at the site of the surgical excision of the mass. The patient complained of increasing fatigue but otherwise was stable with normal blood pressure; examination was remarkable for a soft systolic murmur at the right upper sternal border, mild bilateral lower extremity edema, and elevated jugular venous distension to right mandible with prominent ventricular systolic wave. No other signs of heart failure were identified.

**Figure 1:**
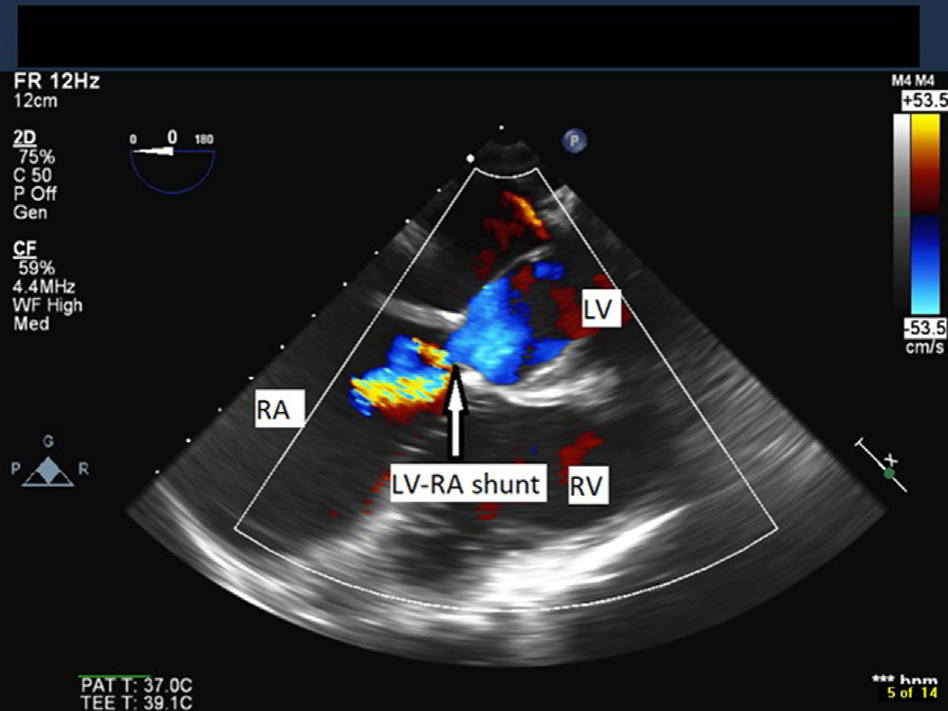
Two-dimensional TEE, midesophageal modified 4-chamber view (0°) with color flow Doppler, systolic phase, demonstrates the LV-RA shunt. *RV*, Right ventricle.

**Figure 2:**
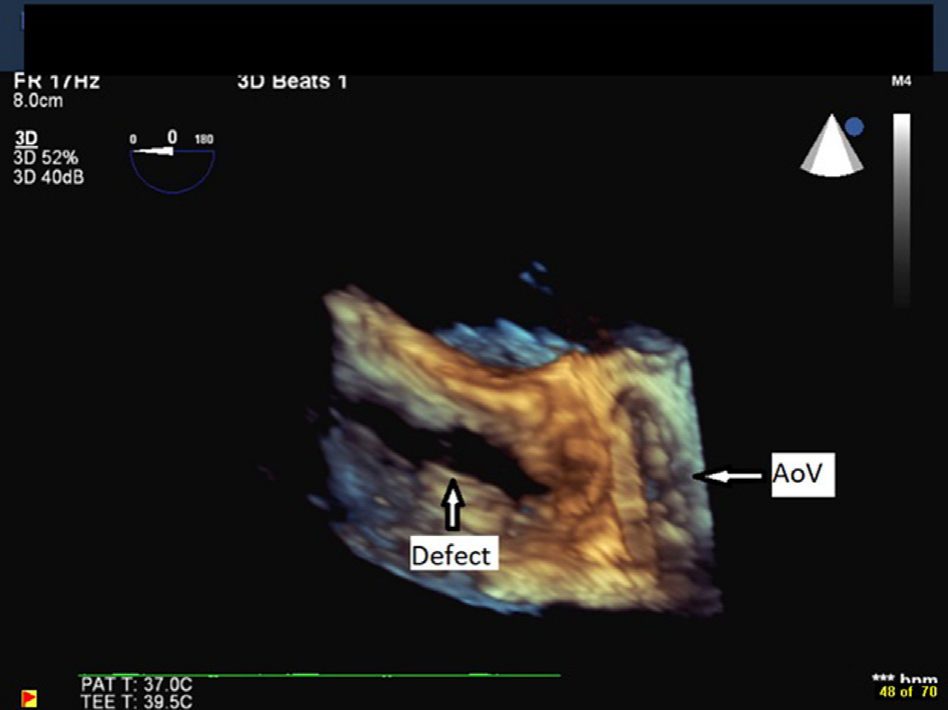
Three-dimensional TEE, midesophageal reconstructed real-time volume-rendered image from the RA perspective demonstrates the Gerbode defect (Defect) in relationship to the aortic valve (AoV).

**Figure 3:**
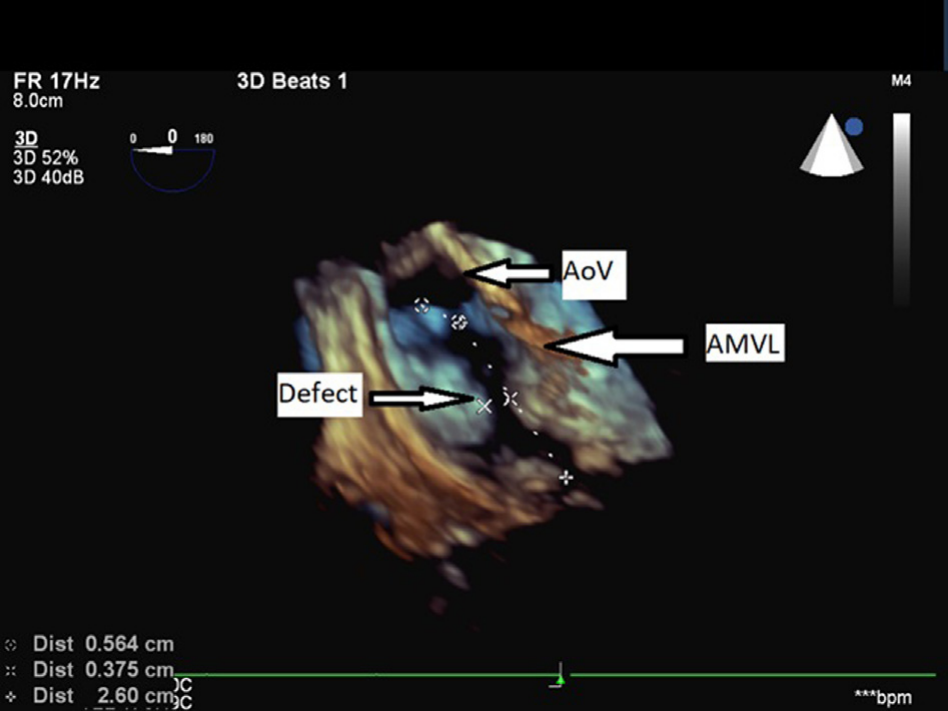
Three-dimensional TEE, midesophageal reconstructed real-time volume-rendered image demonstrates the distance of the Gerbode defect (Defect) from the aortic valve (AoV) annulus. *AMVL*, Anterior mitral valve leaflet.

**Figure 4:**
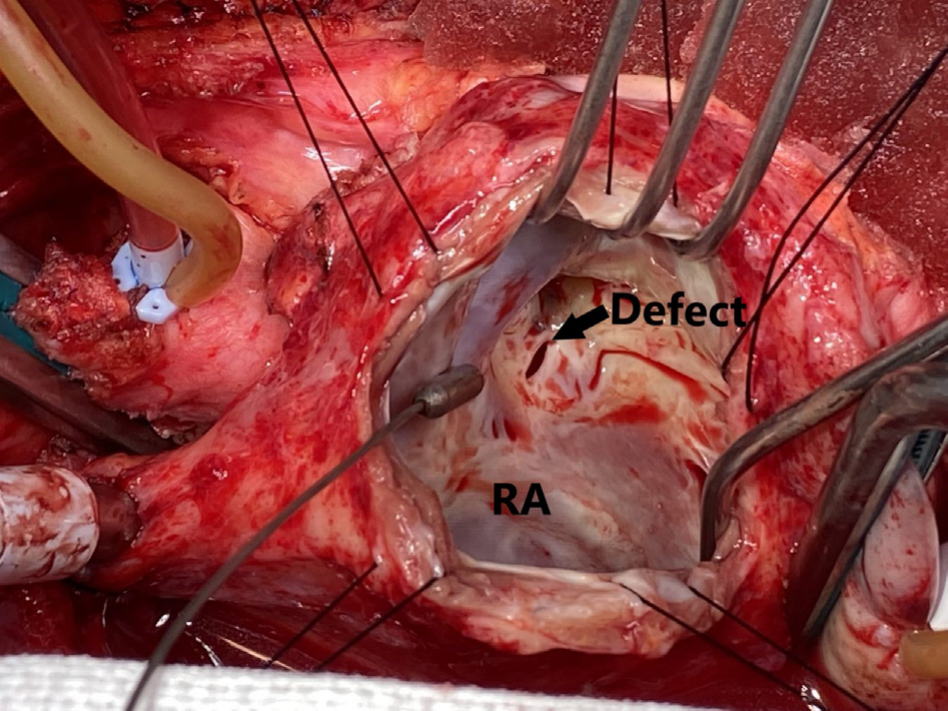
Surgical view of the elongated defect from the right atrial side. Defect indicates the LV-RA communication.

**Figure 5:**
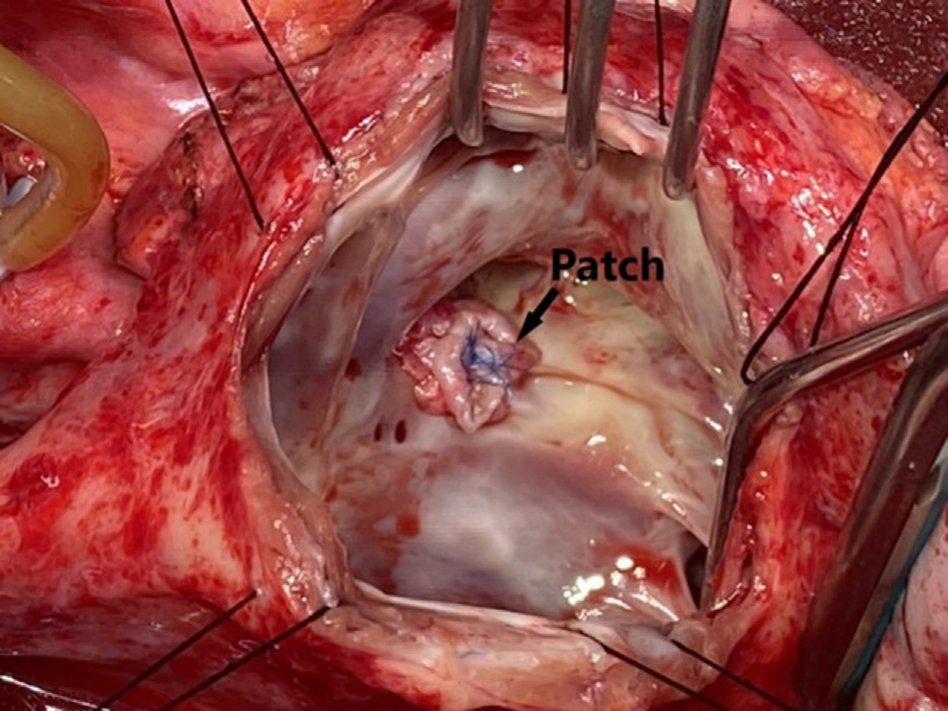
Surgical view after patch closure of the defect as viewed from the right atrial side.

The patient was discussed with the pediatric interventional and cardiovascular surgical team, and a decision was made to attempt to close the defect with a percutaneous device if suitable. With this goal in mind, the patient was brought to the pediatric cardiac catheterization suite. A TEE was ordered to further delineate the anatomy prior to closure. The 2D images showed the LV-RA shunt (Figure 1, Video 1) but could not delineate its relationship to the left ventricular outflow tract and aortic valve. Three-dimensional imaging was performed to further delineate the anatomy (Figures 2 and 3, Video 2). We took care to obtain measurements of the defect with 3D imaging, which are dependent on gain settings that may underestimate or overestimate the size of the actual defect. Since measurements will be affected by parallax and increased slice thickness of 3D rendering, this must be taken into consideration. Acquiring a zoomed or full-volume data set allows these measurements to be accurately obtained using a 3-step approach.^1^ Our measurements demonstrated an elongated defect with a superior anterior margin that was deemed to be too close to the aortic valve for device closure. Hence the patient was referred for surgical closure of the defect. At surgery, the findings were confirmed by direct visualization by surgeons and autologous pericardial patch closure was performed (Figures 4 and 5). The patient was subsequently discharged after an uneventful course on postoperative day 3.

## Discussion

Echocardiography starting with the initial use of M mode and progressing through advances to the current use of 2D, 3D, and 4D imaging of cardiac structures has led to dramatic improvements in cardiovascular medicine and is now the most widely used diagnostic cardiac test after electrocardiography.^2^

Most of the advances have been targeted at assessment of acquired valvular diseases, which predominantly impact adult patients, but these modalities are now being introduced to investigate diseases in pediatric patients and in patients with more complex lesions as well as structural cardiac defects.

Along with the development of the applications, the hardware has also seen significant upgrade over the decades, with the introduction of newer more powerful probes of different sizes, which has led to an exponential growth of echocardiography over the last few decades. Its use has widened to support interventional techniques for percutaneous treatment of structural heart disease^3^ and other interventional procedures.^4^ Almost simultaneously the role of 3D and 4D imaging and its utility in providing additional details that are lacking with 2D echocardiographic imaging have become evident. Its importance in identifying and defining congenital and structural heart defects as well as accurately estimating left and right ventricular function and volume has been discussed.^5^, ^6^, ^7^, ^8^ Kautzner *et al.*^9^ have described the use of 3D and 4D echo application in the electrophysiology lab. They noted that the potential benefits of 3D and 4D echocardiography in electrophysiology lie in real-time guidance of complex ablation procedures and precise assessment of cardiac dyssynchrony.^9^ Roberson and Cui^5^ have elaborately described the use of TEE for ASD closure in the cath lab and have described some of the imaging protocols that they have found useful along with advantages and limitations. With growing experience and confidence in 3D imaging, European Association of Echocardiography/American Society of Echocardiography recommendations for image acquisition and display using 3D echocardiography were published, followed by similar consensus documents for congenital heart diseases.^1^^,^^6^^,^^8^ As experience increases, descriptions of its utility in rarer cardiac diseases are starting to be reported in literature.^7^^,^^10^, ^11^, ^12^

Our case is an attempt to increase the experience in this direction by highlighting the usefulness of the 4D echo to provide additional clinically important information that cannot be gleaned by 2D imaging for structural heart defects. We recommend that 3D echocardiography be fully incorporated in the evaluation of structural cardiac defects. Fortunately, the development in echocardiography is complemented by the development in cardiac computed tomography imaging, CMR imaging, and fluoroscopy (with focus on decreasing fluoroscopy time and rotational angiography). A future direction entails the integration of multiple modalities. Some platforms already allow such integration,^13^ but availability is very limited and integration among all ultrasound platforms is not available. This should be complemented by implementation of a wider use of virtual reality and artificial intelligence. The field seems exciting and full of the potential to lead to better patient outcomes.

## Conclusion

Since its inception, echocardiography has come a long way and has now become the mainstay and workhorse of any cardiac evaluation. The introduction of 3D and 4D imaging has further boosted the utility of this imaging modality. However, weaknesses of 3D imaging remain and have slowed its adoption in congenital and structural heart defects, especially in scenarios where an immediate answer is desired (as in a cardiac interventional suite). Our case highlights that this is achievable and that in select cases it can provide invaluable additional information that can impact management. Hence, its use should be considered in any scenario where 2D imaging is not enough to provide the desired information.

## Ethics Statement

The authors declare that the work described has been carried out in accordance with the following guidelines: This is a case report with no patient identifiers.

## Consent Statement

The authors declare that informed patient consent was not provided for the following reason: this was a non-interventional, retrospective case report utilizing de-identified data, informed consent was not required from the patient as per institutional IRB.

## Funding Statement

The authors declare that this report did not receive any specific grant from funding agencies in the public, commercial, or not-for-profit sectors.

## Disclosure Statement

The authors reported no actual or potential conflicts of interest relative to this document.

## Supplementary Data

Video 1 Two-dimensional TEE, midesophageal modified 4-chamber view (0°) with color flow Doppler demonstrates the LV-RA shunt.

Video 2 Three-dimensional TEE, midesophageal reconstructed real-time volume-rendered image from the RA perspective demonstrates the iatrogenic Gerbode defect in relationship to the aortic valve.
